# Engineering of *E. coli* inherent fatty acid biosynthesis capacity to increase octanoic acid production

**DOI:** 10.1186/s13068-018-1078-z

**Published:** 2018-04-02

**Authors:** Zaigao Tan, Jong Moon Yoon, Anupam Chowdhury, Kaitlin Burdick, Laura R. Jarboe, Costas D. Maranas, Jacqueline V. Shanks

**Affiliations:** 1 Department of Chemical and Biological Engineering, Iowa State University, 3031 Sweeney, Ames, IA 50011 USA; 2 Department of Chemical Engineering, The Pennsylvania State University, University Park, PA 16802 USA

**Keywords:** Computational strain design, OptForce, CRISPR–Cas9, Octanoic acid (C8), Combinatorial engineering

## Abstract

**Background:**

As a versatile platform chemical, construction of microbial catalysts for free octanoic acid production from biorenewable feedstocks is a promising alternative to existing petroleum-based methods. However, the bio-production strategy has been restricted by the low capacity of *E. coli* inherent fatty acid biosynthesis. In this study, a combination of integrated computational and experimental approach was performed to manipulate the *E. coli* existing metabolic network, with the objective of improving bio-octanoic acid production.

**Results:**

First, a customized OptForce methodology was run to predict a set of four genetic interventions required for production of octanoic acid at 90% of the theoretical yield. Subsequently, all the ten candidate proteins associated with the predicted interventions were regulated individually, as well as in contrast to the combination of interventions as suggested by the OptForce strategy. Among these enzymes, increased production of 3-hydroxy-acyl-ACP dehydratase (FabZ) resulted in the highest increase (+ 45%) in octanoic acid titer. But importantly, the combinatorial application of FabZ with the other interventions as suggested by OptForce further improved octanoic acid production, resulting in a high octanoic acid-producing *E. coli* strain +*fabZ* Δ*fadE* Δ*fumAC* Δ*ackA* (TE10) (+ 61%). Optimization of *TE10* expression, medium pH, and C:N ratio resulted in the identified strain producing 500 mg/L of C8 and 805 mg/L of total FAs, an 82 and 155% increase relative to wild-type MG1655 (TE10) in shake flasks. The best engineered strain produced with high selectivity (> 70%) and extracellularly (> 90%) up to 1 g/L free octanoic acid in minimal medium fed-batch culture.

**Conclusions:**

This work demonstrates the effectiveness of integration of computational strain design and experimental characterization as a starting point in rewiring metabolism for octanoic acid production. This result in conjunction with the results of other studies using OptForce in strain design demonstrates that this strategy may be also applicable to engineering *E. coli* for other customized bioproducts.

**Electronic supplementary material:**

The online version of this article (10.1186/s13068-018-1078-z) contains supplementary material, which is available to authorized users.

## Background

The use of microbial biocatalysts for biorenewables production is a promising alternative [1, 2] to the non-renewable oil-based option. Among these biorenewables, fatty acids have received significant attention due to their wide range of applications [3–5]. Fatty acids are directly used a food preservative [4, 5]. They also serve as precursor for synthesis of biocompatible polymers (e.g., polyanhydrides) with low toxicity [6]. In addition to such direct uses, fatty acids can also be described as a chemical intermediate that can be catalytically upgraded to a broad range of chemicals and fuels [7–9]. For instance, fatty acids can be decarboxylated to alkanes as diesel fuel [8, 10], or deoxygenated to alpha-olefins for ethylene polymerization [11, 12]. Furthermore, fatty acids can be converted to fatty acid methyl or ethyl esters (FAME/FAEE), which have higher energy density and lower water solubility than the first-generation biofuel ethanol [13].

Among microbial production organisms, *Escherichia coli* is widely recognized as an excellent host strain for production of biorenewable chemicals or fuels due to its fast growth, sequenced genome, and genetic tractability [12]. A variety of reports have described the metabolic engineering of *E. coli* for production of free fatty acids with high titer and yield [14–19] (Table 1). According to the chain length, fatty acids can be classified as short-chain fatty acids (SCFA), with 6–10 carbons, or medium-chain fatty acids (MCFA), with 12–18 carbons [12]. Octanoic acid (C8) is a representative SCFA and has advantages over MCFA, as octanoic acid methyl or ethyl esters have lower freezing points and kinematic viscosities in contrast to saturated MCFAMEs or MCFAEEs, and thus can enhance fuel quality and have wider applications [20].

**Table 1:** Metabolic engineering of *E. coli* existing fatty acid biosynthesis pathway for fatty acid production in minimal medium

Fatty acid	Strain	Genetic modifications	Thioesterase	Culture condition	Titer (g/L)	Yield (mg/g glucose)	Productivity (mg/L/h)	Source
MCFA (C12–C18)	BL21	Δ*fadL*	TesA’	Bioreactor, minimal medium with 2% (wt/v) glucose	4.8	44	126	[15]
	BL21		AbTE	Bioreactor, M9 medium with 0.5% tryptone and feeding of glucose	3.6	61	89	[19]
	BL21	Δ*fadD*, +*acc*	TesA’ + CcTE	Bioreactor, M9 with feeding of glycerol	2.5	48	170	[16]
	MG1655	Δ*fadD*, +*fabZ*	RcTE	Shake flask, M9 with 1.5% glucose	1.7	113	35	[27]
	DH1	Δ*fadE*	TesA’	Shake flask, minimal with 2% glucose	3.8	190	53	[50]
	DH1	+*fadR*	TesA’	Shake flask, minimal with 2% glucose	5.2	260	72	[51]
	BL21	Modular optimization of multi-genes	CnFatB2	Bioreactor, MK with 1% YE and feeding of glucose	8.6	78	124	[18]
Octanoate (C8)	K27	Δ*fadD*	Various	Culture tube, LB-grown preculture was resuspended in M9 with 0.4% glucose	0.18^a^	48.0^a^	10.0^a^	[24]
	BL21 (DE3)	Δ*fadD* Δ*pta* Δ*lacY fabF*^*mut*^ *fabBDeg*	CpFatB1	96-well plate, LB-grown preculture was diluted 1:20 in M9 with 0.5% glucose	0.24^a^	45.0^a^	4.5^a^	[17]
	MG1655	Δ*fadD*	AtTE	Bioreactor, MOPS with 2% glucose	0.044	4.7	0.45	[26]
	MG1655	*ldhA*::M1-93-*pssA*, *mgsA*::M1-93-*acrAB*, *maeB*::M1-93-*tolC*	AtTE	Shake flask, MOPS with 2% glucose	0.22	20.0	3.1	[25]
	MG1655	*mgsA*::M1-93-*fabZ* Δ*fadE* Δ*fumAC* Δ*ackA*	AtTE	Shake flask, M9 with 1.5% glucose	0.44	30.0	6.2	This study
	MG1655	Same as above	AtTE	Bioreactor, M9 with 1.5% glucose	0.50	33.3	10.4	This study
	MG1655	Same as above	AtTE	Bioreactor, M9 with 2.63% glucose	1.0	38.0	10.4	This study

TE, thioesterase; TesA’, cytosolic *E. coli* TE 1; FatB, plant fatty acyl-ACP thioesterase; Ab, *Acinetobacter baylyi*; Cc, *Cinnamomum camphorum*; Rc, *Ricinus communis*; Cn, *Cocos nucifera*; Cp, *Cuphea palustris*; At, *Anaerococcus tetradius*; YE, yeast extract

^a^Using LB as preculture

Although many efforts have been successful in improving microbial production of MCFA [8, 12, 13, 21–23], production of short-chain C8 is not as advanced. Recently, *S. cerevisiae* and mixed microbial communities have been engineered to produce octanoate; however, the yields were still relatively low (< 320 mg/L) [24–28]. Moreover, to our knowledge, the highest titer in minimal medium of free C8 using *E. coli* engineered with its existing synthesis mechanisms is no more than 250 mg/L [17, 29, 30] (Table 1), which suggests that the existing metabolic network in *E. coli* is not optimal for C8 production. To this end, engineering of *E. coli* inherent metabolism to release its C8 synthesis capacity is desirable. However, engineering strategies that are useful for improving MCFA production might be not simply applied to improving SCFA production due to distinct acyl-ACP thioesterases and optimal rates of acyl-ACP elongation [17, 29]. Although intuitive trial-and-error engineering may be ultimately successful, it is also labor intensive. In contrast, computational strain design tools, such as OptForce, have shown promise in selecting metabolic targets for production of desired targets, due to consideration of the complex interconnectivity of cellular metabolism [12, 31, 32].

**Fig. 1:**
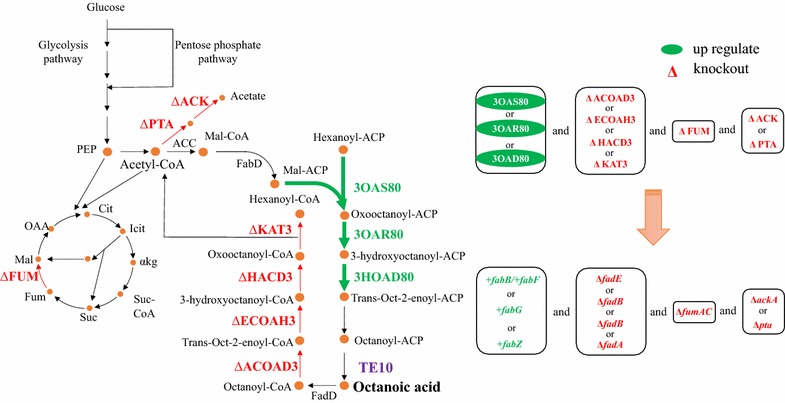
Metabolic interventions suggested by OptForce for increasing production of octanoic acid (C8) in *E*. *coli*. The figure on left shows the reaction-level manipulations in *E*. *coli* central and fatty acid metabolism as suggested by OptForce for overproduction of octanoic acid. Reactions in green are suggested up-regulations (in fatty acid synthesis), while those in red are suggested removals (i.e., in β-oxidation degradation, fumarase, and in the acetate formation pathway). The mapping between reaction-level manipulations to their targeted genes is shown on right. Specifically, 3OAS80, 3-oxy-acyl-ACP synthase encoded by *fabB* or *fabF*; 3OAR80, 3-oxo-acyl-ACP reductase encoded by *fabG*; 3HOAD80, 3-hydroxy-acyl dehydratase encoded by *fabZ*; ACOAD3, acyl-CoA dehydrogenase encoded by *fadE*; ECOAH3, enoyl-CoA hydratase encoded by *fadB*; HACD3, 3-hydroxyacyl-CoA dehydrogenase encoded by *fadB*; KAT3, 3-ketoacyl-CoA thiolase encoded by *fadA*; FUM, fumarase encoded by *fumA* and *fumC*; ACK, acetate kinase encoded by *ackA*; PTA, phosphate acetyltransferase encoded by *pta*

OptForce is an optimization procedure to identify all genetic manipulations required for overproduction of targeted chemicals. It makes use of available flux measurements with a stoichiometric genome-scale model and extracts a minimal set of metabolic fluxes which must actively be forced through genetic manipulations (i.e., FORCE set) to make sure that all fluxes in the metabolic network are consistent with the overproduction objective [33]. Our prior study employed OptForce for prediction of metabolic targets for C8 production [31]. According to the OptForce prediction for C8 overproduction, four distinct interventions (FORCE sets) were suggested (Fig. 1). By using a rapid and iterative CRISPR–Cas9 technique [34], a variety of C8-producing *E. coli* strains were constructed. Among them, the combinatorial ZEFA (TE10) strain exhibits the best performance, resulting in a free C8 titer of 1 g/L (90% extracellular) and a high selectivity (> 70%) in M9 medium using glucose as the sole carbon source. These results demonstrate the effectiveness of combinatorial utilization of computational strain design and experimental approach for free C8 production.

## Results

### Metabolic interventions suggested by OptForce for octanoic acid production

**Fig. 2:**
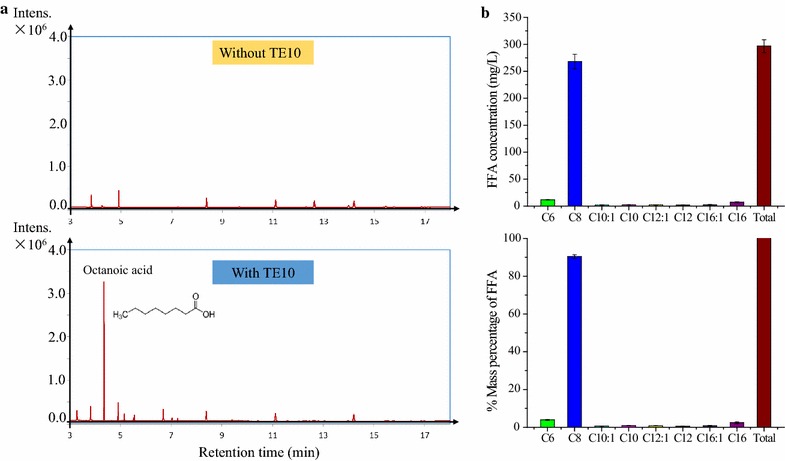
Fatty acids production in *E. coli* strain harboring TE10. **a** Fatty acids profile of *E. coli* MG1655 strains without (upper) and with (lower) pJMYEEI82564 plasmid carrying thioesterase TE10 from *Anaerococcus tetradius*. **b** FFAs concentration and distribution in *E. coli* strain MG1655 harboring TE10. Upper, final free fatty acid titers at 72 h; lower, % mass percentage of produced fatty acids. Cultures were performed in 40 mL M9 + 1.5% (wt/v) dextrose in 250-mL shake flasks at 250 rpm 30 °C with an initial pH of 7.0, IPTG of 1 mM. Titers are the average of at least three biological replicates at 72 h with error bars indicating one standard deviation. TE10, thioesterase from *Anaerococcus tetradius*

The type II fatty acid biosynthesis (FAB) pathway is recognized as the primary route of fatty acid production by *E. coli* [35]. Figure 1 briefly illustrates the anabolic and catabolic metabolism of octanoic acid (C8) within *E. coli*. Acetyl-CoA serves as both initiation primer of fatty acid synthesis and precursor of malonyl-acyl carrier protein (ACP). Specifically, acetyl-CoA is used by acetyl-CoA carboxylase (ACC) to produce malonyl-CoA, which is subsequently converted to malonyl-ACP by malonyl-CoA-ACP transacylase (FabD). The initiation of fatty acid synthesis starts with condensation of acetyl-CoA and malonyl-ACP to form butyryl-ACP, which serves as the core for chain elongation. During each round of the elongation cycle, a two-carbon unit from malonyl-ACP is added to the fatty acyl-ACP chain. Finally, the elongated acyl-ACP is hydrolyzed by thioesterase to release free fatty acid. For production of free C8, the TE10 thioesterase from *Anaerococcus tetradius* [29], which primarily hydrolyzes octanoyl-ACP, was transformed into producer strains. Free octanoic acid (or octanoate anion) whose carboxyl group was not esterified with glycerol-3-phosphate to form lipids is found to be the predominant fatty acid (80–90%) produced by strains harboring *TE10* gene (Fig. 2), which is consistent with previous reports [29]. Conversely, free fatty acids can be also degraded by *E. coli*. Fatty acid is firstly acylated by fatty acyl-CoA synthetase (FadD) to form fatty acyl-CoA, which then enters into the β-oxidation cycle pathway for degradation. During each turn of the oxidation cycle, a two-carbon unit is removed from the acyl-CoA chain to produce one molecule of acetyl-CoA [35].

The OptForce algorithm was previously used to identify a prioritized set of metabolic interventions that would rewire the flux topology of native *E. coli* metabolism towards overproduction of octanoic acid [31, 33] (Fig. 1). The simulations, performed under aerobic glucose minimal medium conditions using the iAF1260 genome-scale metabolic model of *E. coli* (Additional file 1: Table S1) [31], suggested a set of four reaction-level manipulations that improve C8 yield to almost 90% of its theoretical maximum [31, 33] (Fig. 1). The primary interventions include (i) up-regulating any of the fatty acid chain elongation reactions in the C8-chain, i.e., 3-oxy-acyl-ACP synthase (3OAS80), 3-oxo-acyl-ACP reductase (3OAR80), or 3-hydroxy-acyl-ACP dehydratase (3HAD80) by at least two times of the maximum achievable flux in the wild-type *E. coli*; (ii) elimination of any reactions in the β-oxidation pathway, i.e., acyl-CoA dehydrogenase (ACOAD3), enoyl-CoA hydratase (ECOAH3), 3-hydroxyacyl-CoA dehydrogenase (HACD3), 3-ketoacyl-CoA thiolase (KAT3). Together, these two interventions account for over 86% of the predicted C8 yield increase [31].

Besides being the precursor for fatty acid synthesis, acetyl-CoA also has two additional roles. First, acetyl-CoA and oxaloacetic acid (OAA) can be catalyzed by citrate synthase to form citrate as part of the TCA cycle [32]. Consequently, OptForce suggests elimination of fumarase (FUM) to disrupt the TCA cycle activity and maintain a higher pool of acetyl-CoA to be redirected towards the fatty acid elongation. In addition, acetyl-CoA can also be converted by phosphotransacetylase (PTA) and acetate kinase (ACK) to acetate. Therefore, OptForce targets elimination of the acetate formation pathway (Fig. 1). Overall, the flux redirection strategy suggested by OptForce could be summarized as up-regulation of the target pathway (i.e., the C8 chain of fatty acid synthesis), followed by elimination of competing paths that either degrade the product (i.e., the β-oxidation pathway) or degrade the precursor (i.e., the TCA and acetate synthesis pathway). Note that OptForce predictions are based on a stoichiometry-only model of *E. coli* metabolism, and do not include other significant factors, such as enzyme kinetics and transcriptional and substrate-level regulation into consideration. Therefore, the interventions and target yields predicted by OptForce should be considered as a starting point for rewiring the metabolism, rather than guaranteed hits.

### Effects of individual interventions on octanoic acid production

As shown in Fig. 1, interventions suggested by OptForce are at the level of metabolic reactions. In order to implement these interventions genetically, it is essential to translate the “reaction” language into “gene” language [32]. Within the annotated genome of *E. coli* MG1655, we identified a total of 10 genes encoding enzymes for these reactions (Fig. 1). These 10 genes can be divided into four different modules: (i) fatty acids biosynthesis (Fab), including *fabB*, *fabF*, *fabG*, and *fabZ* genes; (ii) fatty acids degradation (Fad), including *fadE*, *fadA*, and *fadB* genes; (iii) FUM, including *fumAC* genes, and (iv) acetate (Ace), including *ackA* and *pta* genes.

The first set of prioritized interventions suggested by OptForce for production of C8 includes up-regulation of one of the chain elongation reactions in the Fab module (Fig. 1). To this end, the effect of increased expression of individual *fab* genes on C8 production was investigated. Prior research has demonstrated that overexpression of pathway genes might cause metabolic burdens, such as from plasmid maintenance, leading to a trade-off in production [36]. To this end, here we overexpressed the *fab* genes by inserting a second copy of *fab* gene into genomic DNA of *E. coli* MG1655 at the *mgsA* site, with regulated by a strong constitutive promoter M1-93 [30]. Increasing the expression of *fabZ*, which encodes the 3-hydroxyacyl-ACP dehydratase, significantly increased C8 production (Table 2). Specifically, +*fabZ* (TE10) strain produced 398 (26.9 mg/g glucose, yield) and 479 mg/L of free C8 and total FAs, which exceeded the wild-type MG1655 (TE10) production of 275 mg/L (19.9 mg/g glucose) free C8 and 315 mg/L total FAs by more than 45 and 52%, respectively (*P* < 0.01). Increased expression of the *fabG* gene, encoding the 3-oxo-acyl-ACP reductase, led to no significant change in C8 production (*P* > 0.05) (Table 2). Unexpectedly, increased expression of some *fab* genes resulted in decreased C8 production. For instance, +*fabB* (3-oxy-acyl-ACP synthase I) (TE10) or +*fabF* (3-oxy-acyl-ACP synthase II) (TE10) strains each produced roughly 250 mg/L of (~ 19.0 mg/g glucose) C8 and 285 mg/L of total FAs, an approximately 10% decrease relative to the starting strain MG1655 (TE10) (*P* < 0.05). While this result is in conflict with the OptForce predictions, which is based only upon stoichiometric considerations, it is consistent with prior observations that inhibiting the activity of FabB and FabF contributed to increased production of C8, likely through regulatory effects [17]. From metabolic control analysis, linear pathway enzymes may share control for the flux through that pathway, and thus genes for all enzymes in the metabolic pathway should be overexpressed together for further increasing the production of desired targets [37–41]. To this end, +*fabG* and +*fabB/F* based on +*fabZ* was performed (+*fabZGB/*+*fabZGF*, Additional file 2: Figure S1). However, compared with +*fabZ*, both +*fabZGB* and +*fabZGF* decreased rather than increased C8 production (Table 2).

**Table 2:** Effects of engineering individual genes on free octanoic acid production

Strain	Time (h)	Cell density (OD_550_)	Glucose used (g/L)	Acetate titer (g/L)	C8 titer (mg/L)	C8 yield (mg/g glucose)	C8 productivity (mg/L/h)	Total FFAs (mg/L)
MG1655 (TE10)	72	1.91 ± 0.1	13.8 ± 0.2	0.63 ± 0.02	275 ± 12	19.9 ± 0.9	3.81 ± 0.17	315 ± 14
+*fabB* (TE10)	72	1.87 ± 0.06	13.4 ± 0.1	0.62 ± 0.02	250 ± 5.7	18.6 ± 0.4	3.46 ± 0.079	281 ± 5.7
+*fabF* (TE10)	72	1.72 ± 0.05	13.2 ± 0.3	0.65 ± 0.03	253 ± 6.4	19.2 ± 0.3	3.52 ± 0.088	292 ± 11
+*fabG* (TE10)	72	1.81 ± 0.1	13.9 ± 0.4	0.67 ± 0.05	293 ± 5.5	21.0 ± 0.4	4.07 ± 0.076	326 ± 28
+*fabZ* (TE10)	72	4.51 ± 0.2	14.8 ± 0.1	1.8 ± 0.2	398 ± 8.3	26.9 ± 0.6	5.53 ± 0.12	479 ± 22
+*fabZGB* (TE10)	72	5.74 ± 0.4	14.8 ± 0.1	0.52 ± 0.06	278 ± 13	18.8 ± 0.9	3.86 ± 0.18	403 ± 16
+*fabZGF* (TE10)	72	5.50 ± 0.2	14.6 ± 0.1	1.1 ± 0.2	207 ± 9.5	14.2 ± 0.7	2.88 ± 0.13	309 ± 11
Δ*fadE* (TE10)	72	2.20 ± 0.1	13.6 ± 0.4	1.2 ± 0.1	286 ± 8.8	21.1 ± 0.6	3.97 ± 0.12	333 ± 10
Δ*fadB* (TE10)	72	1.84 ± 0.1	13.3 ± 0.1	1.3 ± 0.1	270 ± 4.4	20.3 ± 0.3	3.75 ± 0.062	310 ± 5.2
Δ*fadA* (TE10)	72	1.84 ± 0.02	13.8 ± 0.2	1.4 ± 0.1	270 ± 3.5	19.5 ± 0.3	3.74 ± 0.048	309 ± 4.4
Δ*fumAC* (TE10)	72	1.73 ± 0.07	12.9 ± 0.3	1.4 ± 0.2	241 ± 6.5	18.7 ± 0.5	3.35 ± 0.048	277 ± 3.9
Δ*ackA* (TE10)	72	2.06 ± 0.06	13.2 ± 0.1	0.6 ± 0.1	289 ± 5.7	21.9 ± 0.4	4.00 ± 0.079	330 ± 6.6
Δ*pta* (TE10)	72	1.97 ± 0.03	12.9 ± 0.2	0.5 ± 0.1	284 ± 2.6	22.0 ± 0.2	3.94 ± 0.036	324 ± 3.4

In this study, the genetic interventions identified by OptForce are prioritized based on their impact on C8 product yield improvement. In addition to the first set, the second set of prioritized interventions predicted by OptForce is interruption of the β-oxidation cycle. In contrast to the first set of Fab module, engineering of the corresponding Fad module by deletion of any *fadE* (acyl-CoA dehydrogenase), *fadA* (3-ketoacyl-CoA thiolase), or *fadB* (enoyl-CoA hydratase/3-hydroxyacyl-CoA dehydrogenase) led to no significant change in production of C8 or total FAs (*P* > 0.05) (Table 2).

The third set of prioritized interventions suggested by OptForce is to remove fumarase (FUM) in the TCA cycle in order to maintain higher acetyl-CoA pool towards the C8 elongation chain. *E. coli* has three distinct fumarase isozymes, encoded by *fumA*, *fumB*, and *fumC* [42]. Unlike *fumB*, the *fumA* and *fumC* genes are expressed primarily under aerobic conditions [43]. Therefore, *fumA* and *fumC* were selected as targets for disruption of aerobic fumarase activity. Since *fumA* and *fumC* are co-transcribed, the two genes were deleted simultaneously, resulting in Δ*fumAC* strain. Results showed that Δ*fumAC* (TE10) decreased free C8 and total FAs production by 12% to 241 mg/L (18.7 mg/g glucose) and 277 mg/L relative to the starting strain MG1655 (TE10) (*P* < 0.01) (Table 2). The fourth set of prioritized interventions involves removal of phosphotransacetylase (PTA) and acetate kinase (ACK) in the acetate formation pathway (Ace module). Specifically, individual deletion of *ackA* or *pta* resulted in limited change in free C8 or total FAs production (Table 2), which is consistent with the prior observation of deletion effects on long-chain fatty acid production [44].

Taken together, among these interventions, engineering of the Fab module, especially the up-regulation of *fabZ* (+*fabZ*), imposed the highest improvement in C8 production, which is in line with the prioritized order suggested by OptForce.

### Combinatorial utilization of the suggested four interventions

While there are cases where individual interventions have improved the target yield [32], in this case for C8, OptForce suggests these interventions should be examined in their prioritized order. Actually, effects of modules with less priority such as FUM, Ace should be seen in conjunction with modules with high priority, such as Fab and Fad modules. Therefore, after knowing the effect of individual interventions on C8 production, combinatorial utilization of interventions was performed with the aim of further C8 improvement, and the CRISPR–Cas9 method, which has the advantages of iterative genome engineering, was further employed to construct these combinatorial strains [34].

**Fig. 3:**
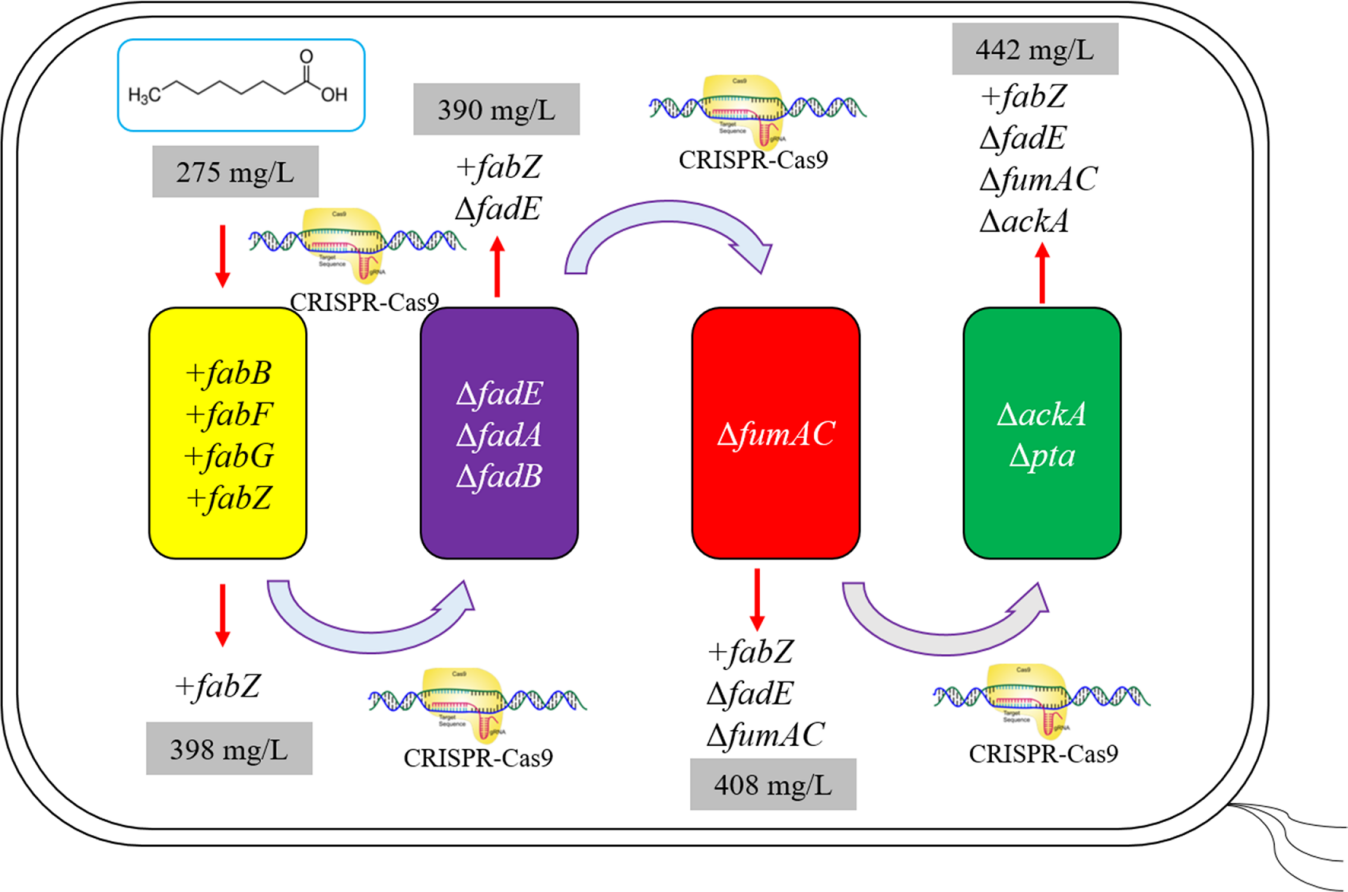
Schematic of combinatorial utilization of different interventions for octanoic acid production. CRISPR–Cas9 technique [34] was employed to construct these combinatorial engineered strains

As the +*fabZ* intervention in the Fab module enabled the highest increase in C8 production (+ 45%) (Table 2; Fig. 3), it was selected as the representative of the Fab module for subsequent implementation of other interventions. The suggested genes in the Fad module (*fadE*, *fadB*, *fadA*) were then individually deleted from the +*fabZ* (TE10) strain. However, no significant increase was observed in free C8 production (Table 3). Since +*fabZ* Δ*fadE* (TE10) was one of the best-performing strains after +*fabZ* Δ*fad* engineering, it was therefore selected for the next round of FUM module engineering.

**Table 3:** Effects of combinatorial implementation of different interventions on free octanoic acid production

Strain	Time (h)	Cell density (OD_550_)	Glucose used (g/L)	Acetate titer (g/L)	C8 titer (mg/L)	C8 yield (mg/g glucose)	C8 productivity (mg/L/h)	Total FFAs (mg/L)
MG1655 (TE10)	72	1.91 ± 0.1	13.8 ± 0.2	0.63 ± 0.02	275 ± 12	19.9 ± 0.9	3.81 ± 0.17	315 ± 14
+*fabZ* (TE10)	72	4.51 ± 0.2	14.8 ± 0.1	1.8 ± 0.2	398 ± 8.3	26.9 ± 0.6	5.53 ± 0.12	479 ± 22
+*fabZ* Δ*fadE* (TE10)	72	4.62 ± 0.1	14.8 ± 0.1	1.7 ± 0.02	387 ± 12	26.2 ± 0.8	5.38 ± 0.16	462 ± 14
+*fabZ* Δ*fadB* (TE10)	72	4.76 ± 0.3	14.8 ± 0.1	1.3 ± 0.3	395 ± 8.2	26.7 ± 0.5	5.49 ± 0.11	469 ± 12
+*fabZ* Δ*fadA* (TE10)	72	1.90 ± 0.1	13.2 ± 0.4	0.9 ± 0.2	300 ± 5.7	22.7 ± 0.4	4.17 ± 0.079	350 ± 6.2
+*fabZ* Δ*fadE* Δ*fumAC* (TE10)	72	4.74 ± 0.2	14.4 ± 0.2	1.1 ± 0.2	408 ± 4.8	28.3 ± 0.3	5.67 ± 0.066	515 ± 8.8
+*fabZ* Δ*fadE* Δ*fumAC* Δ*ackA* (TE10)	72	5.38 ± 0.1	14.8 ± 0.1	1.4 ± 0.1	442 ± 14	30.0 ± 0.8	6.15 ± 0.19	615 ± 21
+*fabZ* Δ*fadE* Δ*fumAC* Δ*pta* (TE10)	72	4.79 ± 0.03	14.6 ± 0.1	1.4 ± 0.3	408 ± 4.9	27.2 ± 0.3	5.67 ± 0.068	532 ± 5.9

In contrast to the decreased C8 production by the Δ*fumAC* (TE10) strain, disruption of *fumAC* in the +*fabZ* Δ*fadE* (TE10) strain improved free C8 production (Table 3). Specifically, the triple +*fabZ* Δ*fadE* Δ*fumAC* (TE10) produced 408 mg/L of C8 (28.3 mg/g glucose) and 515 mg/L of total FAs, which exceeds that of +*fabZ* Δ*fadE* (TE10) strain (C8 = 387 mg/L, 26.2 mg/g glucose; total FAs = 462 mg/L) by 5.4 and 11.5%, respectively (*P* = 0.04). This observation is in line with OptForce predictions that suggest an increase in octanoic acid yield due to removal of fumarase activity only in the background of manipulations in the fatty acid synthesis and degradation pathways. This +*fabZ* Δ*fadE* Δ*fumAC* (TE10) strain was used for the next round engineering of Ace module engineering. Although deletion of *pta* in +*fabZ* Δ*fadE* Δ*fumAC* (TE10) did not further increase free C8 production, deletion of *ackA* did substantially improve C8 production (*P* = 0.009) (Fig. 3). Specifically, the resulting quadruple strain +*fabZ* Δ*fadE* Δ*fumAC* Δ*ackA* (TE10) (ZEFA (TE10)) produced 442 mg/L of C8 (30.0 mg/g glucose) and 615 mg/L of total FAs, which exceeded that of +*fabZ* Δ*fadE* Δ*fumAC* (TE10) by approximately 10 and 20% (C8 = 408 mg/L, 28.3 mg/g glucose; total FAs = 515 mg/L) (Table 3; Fig. 3). Compared with the starting strain +*fabZ* (TE10) (C8 = 398 mg/L, 26.9 mg/g glucose; total FAs = 479 mg/L), ZEFA (TE10) produced 11 and 28% more C8 and total FAs (*P* = 0.002). Compared with the wild-type MG1655 (TE10) (C8 = 275 mg/L, 19.9 mg/g glucose; total FAs = 315 mg/L), ZEFA (TE10) produced 61 and 95% more C8 and total FAs (*P* < 0.001) (Table 3; Fig. 3). Similar to deletion of Δ*fumAC,* this result also highlights the prioritized intervention strategy suggested by OptForce for overproduction of octanoic acid.

### Optimization of culture conditions for octanoic acid production

**Fig. 4:**
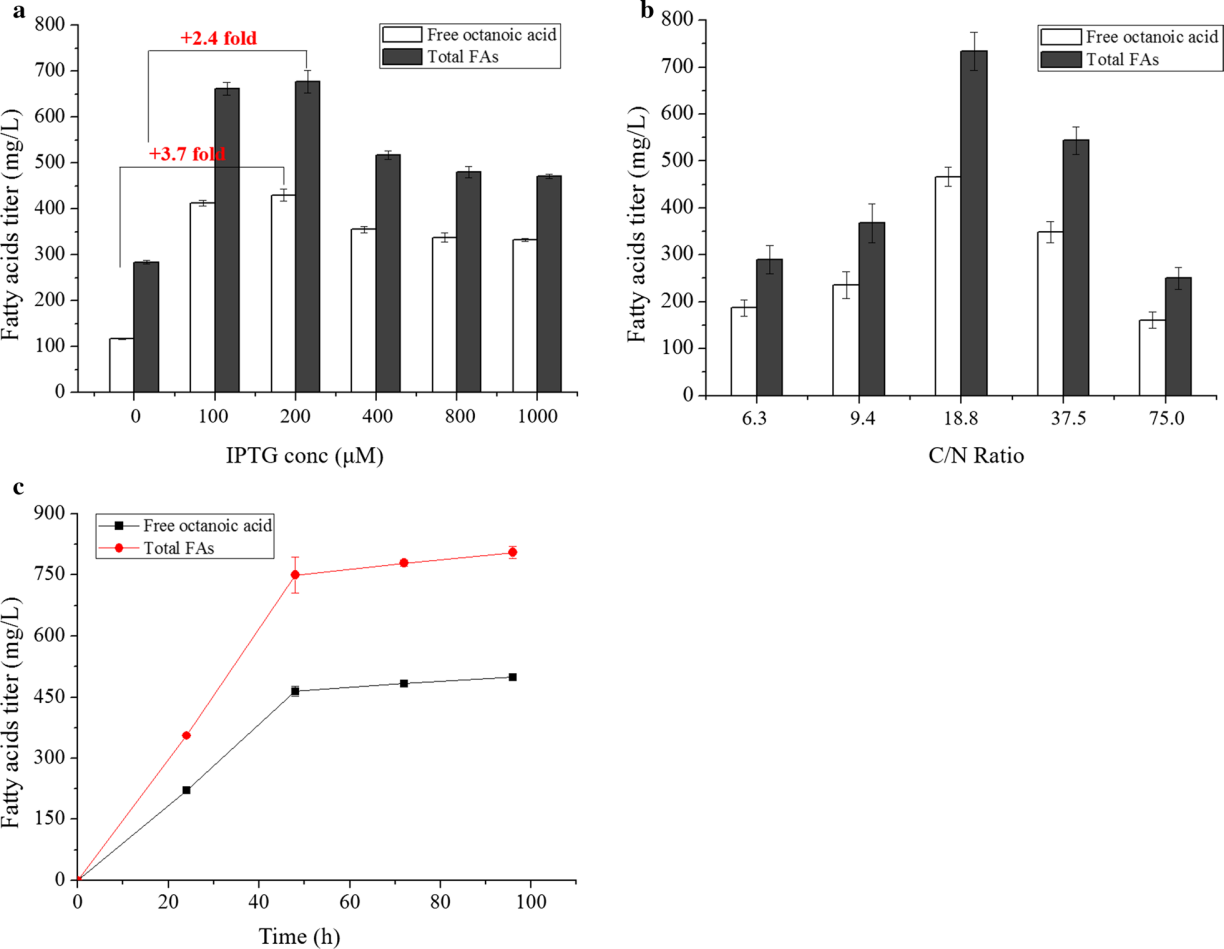
Optimization of culture conditions for free octanoic acid production in the ZEFA (TE10) strain. **a** Effects of different IPTG dosages on octanoic acid production. ZEFA (TE10) strain produced the highest titer of octanoic acid (430 mg/L) when induced with 200 μM IPTG for increasing expression of thioesterase *TE10* gene. Cultures were performed in 40 mL M9+ 1.5% (wt/v) dextrose with the C/N ratio of 18.8 in 250-mL shake flasks at 250 rpm 30 °C with an initial pH of 7.0 and the IPTG was added when the OD_550_ reached 0.4–0.5. **b** Effects of different initial C/N ratios on octanoic acid production under 200 μM IPTG. ZEFA (TE10) strain has the highest C8 production at the C/N ratio of 18.8. For changing the C/N ratio, the glucose (carbon source) concentration is fixed at 15 g/L and the amount of NH_4_Cl (carbon source) added was varied accordingly. (C) Maintenance of culture broth pH at neutral range (pH = 7.0) increased octanoic acid production under 200 μM IPTG and C/N ratio of 18.8. Culture was performed in 300 mL M9+ 1.5% (wt/v) dextrose in a 500-mL bioreactor. Cultures were grown at 30 °C, and the pH was maintained at 7.0 by adding 2.0 M potassium hydroxide (KOH). Air flow rate was controlled at 0.3 L/min with 300 rpm as the initial stirring speed. The dissolved oxygen (DO) level was set over 40% and controlled by changing the stirring speed with the maximum of 600 rpm. Titers are the average of at least three replicates with error bars indicating one standard deviation. ZEFA, +*fadZ* Δ*fadE* Δ*fumAC* Δ*ackA*

In this study, gene for encoding thioesterase TE10 is expressed under the control of trc promoter, and thus IPTG serves as inducer for expression of *TE10* gene and C8 production. However, although useful, excessive IPTG addition has been reported to be toxic to *E. coli* cells [45], resulting in inhibition of enzymatic activities and decreased product biosynthesis [46]. To this end, optimization of IPTG dosage for fatty acid production is desirable [47]. Since ZEFA (TE10) is the best-performing C8-producing strain obtained in this study, it was selected for IPTG dosage optimization. Results showed that the ZEFA (TE10) strain can still produce 117 mg/L of free C8 without addition of IPTG, which means there is leaky expression of *TE10* gene under the trc promoter (Fig. 4a). Upon induction by IPTG, C8 titer elevated significantly and a positive correlation was observed between IPTG dosage and C8 titer up to 200 μM. At the dosage of 200 μM IPTG, ZEFA (TE10) strain produced 430 mg/L of C8 and 678 mg/L of total FAs, which was 3.7- and 2.4-fold of the non-IPTG condition (Fig. 4a). However, excessive dosage of IPTG, e.g., > 200 μM, was found to decrease C8 production (Fig. 4a).

The carbon-to-nitrogen (C/N) ratio has been observed to affect the production of biomass and fatty acid production in some microorganisms [48]. In order to investigate the impact of the C/N ratio on free C8 production in the ZEFA (TE10) strain, we varied the initial C/N ratio in the M9 medium from 6.25 to 75.0 (Fig. 4b). Results showed that ZEFA (TE10) produced the highest C8 titer of 466 and 730 mg/L of total FAs (IPTG = 200 μM) at the C/N ratio of 18.8, and either lower or higher C/N ratio was found to compromise C8 production (Fig. 4b).

In addition, we also found that medium acidification might be another limiting factor for free C8 production. For instance, the final pH of ZEFA (TE10) culture broth in shake flasks at 72 h was approximately 5.5 (data not shown). Broth acidification can inflict detrimental effects to *E. coli* growth and activities of key metabolic enzymes [49]. Maintenance of pH at neutral range is expected to mitigate the acidification burden and thus possibly improve C8 production. To this end, a pH-controlled bioreactor, instead of shake flasks, was employed. With this pH control, the ZEFA (TE10) strain produced 500 mg/L of C8 and 805 mg/L of total FAs (pH = 7.0, IPTG = 200 μM), which is a 7.3 and 10.3% increase relative to shake flasks (C8 = 466 mg/L; TFA = 730 mg/L) (*P* < 0.05) (Fig. 4c), and a 82 and 155% increase relative to wild-type MG1655 (TE10) in shake flasks (C8 = 275 mg/L; total FAs = 315 mg/L) (*P* < 0.01).

### Fed-batch culture of strain ZEFA (TE10) for octanoic acid production

**Fig. 5:**
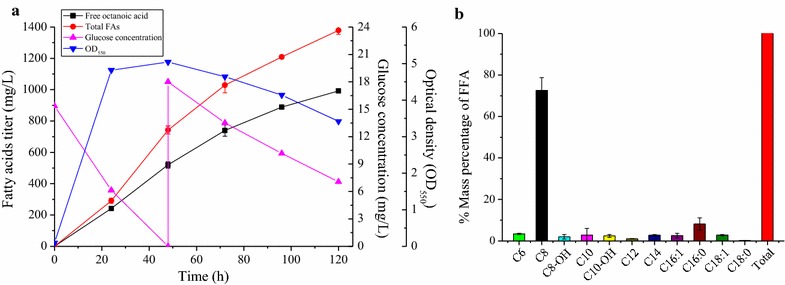
Fed-batch culture of strain ZEFA (TE10) for free octanoic acid production. **a** Profile of fed-batch culture. **b** FFAs distribution in ZEFA (TE10) at the end of culture. Culture was initially performed in 300 mL M9+ 1.5% (wt/v) dextrose in 500-mL bioreactor. An additional 9 mL of fresh 50% (wt/v) glucose was added after 48 h, as indicated. Cultures were grown at 30 °C, and the pH was maintained at 7.0 using 2 M potassium hydroxide (KOH). Air flow rate was controlled at 0.3 L/min with 300 rpm as the initial stirring speed. The dissolved oxygen (DO) level was set over 40% and controlled by changing the stirring speed with the maximum of 600 rpm. Octanoic acid titers are the average of at least three replicates with error bars indicating one standard deviation. ZEFA, +*fadZ* Δ*fadE* Δ*fumAC* Δ*ackA*

In this study, M9 medium with 1.5% (wt/v) glucose was used as culture broth for free C8 production. For the ZEFA (TE10) strain, we found that there was no residual glucose after 72-h cultivation in shake flasks (data not shown). Since glucose is the substrate, the provisioning of additional glucose may increase C8 titers. To this end, fed-batch culture was performed to the ZEFA (TE10) strain with previously described optimized culture conditions, i.e., pH maintained at 7.0, IPTG added at 200 μM, and the initial C/N ratio kept at 18.8. Initially, 15 g/L of glucose was used as carbon source and another 15 g/L of fresh glucose was fed after 48-h cultivation (Fig. 5). Results showed that cell growth of the ZEFA (TE10) reached stationary phase at 24 h, and the final highest optical density (OD_550_) achieved 5.18 at 48 h. The total culture process lasted for 120 h and the ZEFA (TE10) consumed a total of 26.3 g/L of glucose, produced approximately 1 g/L of free C8 (38.5 mg/g glucose, 11% maximum theoretical yield) and 1.4 g/L of total FAs (Fig. 5). Furthermore, over 90% of the C8 is recovered in the medium (data not shown). To our knowledge, this is the highest free C8 titer achieved in minimal medium using *E. coli* inherent fatty acid biosynthesis pathway.

## Discussion

In this study, we systematically engineered *E. coli* inherent metabolism network for overproduction of free C8. Different from previous strategies, this work focuses on exploiting rational computational design to identify the metabolic targets. Finally, a total of ten enzymes in four different interventions (or modules) are suggested by OptForce to be manipulated production. Here we found the effects of manipulations on C8 production are in line well with OptForce predictions of prioritizing orders. Specifically, up-regulation of the Fab module is suggested to impose the most promising effect on C8 yield improvement and the experimental result confirmed this conclusion: increased expression of *fabZ* enabled the highest increase of C8 titer (+ 45%) in +*fabZ* (TE10) engineered strain. Although this study focuses on C8 production, the potential positive effect of +*fabZ* intervention was also observed in other cases, such as MCFA (C14–C16) production in *E. coli* [23]. In addition, in vitro titration experiment also showed that addition of purified FabZ enzyme exhibits the highest increase in the rate of FAS reaction among different FAS subunits [50], which agrees with the effectiveness of +*fabZ* and the utility of the OptForce prediction. Although +*fabG* has been shown effective in production of MCFA [51], it exhibits limited effect on C8 production in this study, which suggests the differences underlying MCFA and C8 biosynthesis.

In contrast to +*fabZ*, +*fabB* and +*fabF* were found to decrease C8 production and total fatty acids to some extent. Both +*fabB* and +*fabF* have been reported to speed up the acyl-ACP elongation, resulting in depletion of C8-ACP levels for C8 production; furthermore, the longer acyl-ACPs feedback inhibits the initiation step of fatty acid biosynthesis [17]. In an attempt to overcome this feedback inhibition by overexpressing *fabZ* together with *fabG* and *fabB*/*fabF*, +*fabG* and +*fabB/F* strains based on +*fabZ* were constructed and analyzed for fatty acid production (+*fabZGB/*+*fabZGF*, Additional file 2: Figure S1). The overexpression of *fabZGB* (+*fabZGB*) increased C8 and total fatty acid over the wild type but +*fabZGF* did not. Importantly, compared with +*fabZ*, both +*fabZGB* and +*fabZGF* decreased rather than increased C8 production and total fatty acid production (Table 2). Prior study of dynamic manipulation of FabB and FabF did result in enhanced production of C8 over the wild type in minimal media [17] but FabZ was not considered in that study. The complex regulation in this recursive biosynthetic system presents a challenge for optimizing C8 relative to total fatty acid production at the higher titers needed for industrial production.

Individual intervention of genes in Fad module, FUM module, and Ace module shows no significant positive or even negative effects on C8 production (Table 2). It is consistent with the prioritizing orders of OptForce predictions: all of these interventions should be seen effectiveness for C8 production when in conjunction with the Fab module. Δ*fumAC* alone was found to moderately compromise cellular growth and C8 production (Table 2). One possibility is that Δ*fumAC* alone will cause excessive accumulation of acetyl-CoA for compromised TCA cycle which is the main sink for acetyl-CoA catabolic metabolism. In this study, we observed that +*fabZ/*Δ*fadE* manipulations based on Δ*fumAC* instead significantly increased cellular growth and C8 production (Additional file 3: Figure S2). This phenomenon can be explained well by +*fabZ* which effectively channels excessive acetyl-CoA into C8 biosynthesis and Δ*fadE* also alleviates further accumulation of acetyl-CoA sourcing from fatty acid degradation. In addition, since the TCA cycle serves as a primary source for ATP and NAD(P)H factors, compromised TCA cycle activity from Δ*fumAC* might impair cell energy metabolism [52]. Prior studies found that increasing ATP energy supply improved fatty acid production under high cell density condition [53]. Hence, balancing the flux between entering into cell energy metabolism and fatty acid biosynthesis should be cautiously considered.

After combinatorial utilization of Fad, FUM, and Ace modules based on Fab module according to the suggested prioritizing order, we finally obtained the best C8 production strain *E. coli* ZEFA (TE10). Overall, the manipulations of +*fabZ*, Δ*fadE*, Δ*fumAC*, and Δ*ackA* in ZEFA (TE10) have a synergistic role in increasing C8 production: +*fabZ* primarily strengthens C8 biosynthesis pathway efficiency, Δ*fadE* eliminates β-oxidation of C8 degradation, and Δ*fumAC* in the TCA cycle and Δ*ackA* in acetate formation maintain a higher acetyl-CoA pool for channeling into C8 biosynthesis pathway strengthened by +*fabZ*. All of these interventions enabled the ZEFA (TE10) strain to produce 1 g/L of free C8 in M9 medium, which is the highest titer under minimal medium using *E. coli* native fatty acid biosynthesis mechanism to our knowledge (Table 1).

It is to be noted that OptForce does not suggest the increased production of acetyl-CoA carboxylase (ACC) for C8 production, which is supposed to drive more acetyl-CoA flux to malonyl-CoA for C8 biosynthesis. In order to investigate the effect of increasing production of ACC on C8 production, +*accABCD* was performed in our engineered strain. However, no improvement in free C8 production was observed (Additional file 4: Figure S3), indicating that acetyl-CoA carboxylation might be not one of the bottlenecks for C8 production. This result is consistent with previous observations that increased production of ACC did not lead to significant increase in production of MCFA [8, 16].

In this study, besides genetic interventions, we also optimized the culture conditions for C8 production, e.g., IPTG dosage, C/N ratio, and control of pH at neutral range, all of which were found to contribute to free C8 production. However, although C8 titer and yield in ZEFA (TE10) strain after four rounds of engineering increased significantly compared with the starting strain, it is still lower than the predicted result of OptForce. Specifically, OptForce predicted to yield almost 90% of the theoretical maximum of C8 production (~ 0.34 g/g glucose), while only 11% (0.0385 g/g glucose) was experimentally achieved in ZEFA (TE10). OptForce predictions are solely based on stoichiometric limitations with no underlying kinetic and regulatory constraints, while these factors are significant contributors towards limiting carbon flux [31, 33]. In addition, low catalytic capacity of thioesterase TE10 used here for hydrolysis of octanoyl-ACP, the complex regulation of the recursive fatty acid biosynthesis pathway which complicates the balance of C8 and longer chain fatty acids at high titers, and importantly, toxicity from the end-product of C8 might also compromise strain performance and thus C8 titer and yield [49, 54]. Prior research has shown that octanoic acid toxicity increases at lower pH values, particularly as the media pH nears the molecule pKa [49, 54]. Considering that membrane damage is deemed as a fundamental mechanism of fatty acid toxicity, and membrane engineering has proven its effectiveness in alleviating membrane damage from fatty acids [30, 55–58], it is reasonable to expect that application of these membrane engineering strategies combined with the metabolic interventions here will contribute to further C8 improvement in the future.

Recent research about biosynthesis of SCFA (C6–C10, they defined as MCFA there) in *E. coli* via introduction of the non-native reverse beta-oxidation cycle (r-BOX) has led to titers of > 1 g/L in rich medium [59]. Heterologous thiolases and trans-enoyl-CoA reductase in conjunction with endogenous FadB and thioesterase were activated for operation of r-BOX. Although a high titer of SCFA in rich medium was achieved (3.8 g/L), the SCFA was a mixture of C4, C6, C8, and C10. Due to the r-BOX inherent characteristic of operation in an iterative manner [14, 60, 61], it may be challenging to narrow down the MCFA to a specific length. In this study, we selectively produced C8 from the other fatty acids, which then enables more efficient recovery of the desired product.

In summary, by implementing a minimal set of metabolic interventions, we have successfully engineered *E. coli* MG1655 inherent metabolism network and achieved the high production and selectivity of C8. Besides C8, this strategy which integrates computational strain design and experimental study is expected to be applicable to production of other biorenewables.

## Conclusions

In this study, a combination of integrated computational and experimental approach was performed to manipulate the *E. coli* native metabolic network for octanoic acid (C8) production. Four interventions were subsequently predicted by a customized OptForce methodology. Then, all the ten associated candidate proteins were regulated individually and combinatorially. +FabZ was identified as the most prominent individual intervention and the final combinatorial strain based on +FabZ eventually produced 1 g/L of C8 with high selectivity in minimum medium using glucose as single carbon. This work underlines the significance of integration of computational strain design and experimental testing in rewiring microbial metabolism for octanoic acid production. This result besides other studies using OptForce in strain design demonstrates that this strategy may be also applicable to engineering *E. coli* for production of other customized biorenewable chemicals or biofuels.

## Methods

### Strains and plasmids

All plasmids and strains used in this study are listed in Table 4. Primers can be found in Additional file 5: Table S2. *E. coli* K-12 MG1655 was used as the base strain in this study. The scarless CRISPR–Cas9 approach was employed to perform genetic deletion and tuning gene expression [34]. For increasing expression of *fab* genes (*fabB*, *fabF*, *fabG*, *fabZ*), a second copy of each gene with a strong constitutive M1-93 promoter [30, 58] was inserted into chromosomal DNA of MG1655 at *mgsA* site. For octanoic acid production, the pJMYEEI82564 plasmid [30, 62], harboring the *Anaerococcus tetradius* thioesterase (TE10) that primarily hydrolyzes octanoyl-ACP [29], was transformed into our engineered strains. When necessary, ampicillin, kanamycin, and chloramphenicol were used at a final concentration of 100, 50, and 34 mg/L, respectively.

**Table 4:** Strains and plasmids used in this study

Strains/plasmids	Genetic characteristics	Source
Strains		
MG1655	Wild-type *E. coli* K1-12 strain	Lab collection
+*fadB*	MG1655, *mgsA*::M1-93-*fadB*	This study
+*fadF*	MG1655, *mgsA*::M1-93-*fadF*	This study
+*fadG*	MG1655, *mgsA*::M1-93-*fadG*	This study
+*fadZ*	MG1655, *mgsA*::M1-93-*fadZ*	This study
+*fadZGB*	MG1655, *mgsA*::M1-93-*fadZ*-*RBS1*-*fabG*-*RBS2*-*fabB*	This study
+*fadZGF*	MG1655, *mgsA*::M1-93-*fadZ*-*RBS1*-*fabG*-*RBS2*-*fabF*	This study
Δ*fadE*	MG1655, Δ*fadE*	This study
Δ*fadA*	MG1655, Δ*fadA*	This study
Δ*fadB*	MG1655, Δ*fadB*	This study
Δ*fumAC*	MG1655, Δ*fumAC*	This study
Δ*ackA*	MG1655, Δ*ackA*	This study
Δ*pta*	MG1655, Δ*pta*	This study
+*fadZ* Δ*fadE*	MG1655, *mgsA*::M1-93-*fadZ*, Δ*fadE*	This study
+*fadZ* Δ*fadA*	MG1655, *mgsA*::M1-93-*fadZ*, Δ*fadA*	This study
+*fadZ* Δ*fadB*	MG1655, *mgsA*::M1-93-*fadZ*, Δ*fadB*	This study
+*fadZ* Δ*fadE* Δ*fumAC*	MG1655, *mgsA*::M1-93-*fadZ*, Δ*fadE*, Δ*fumAC*	This study
+*fadZ* Δ*fadE* Δ*fumAC* Δ*ackA* (ZEFA)	MG1655, *mgsA*::M1-93-*fadZ*, Δ*fadE*, Δ*fumAC*, Δ*ackA*	This study
+*fadZ* Δ*fadE* Δ*fumAC* Δ*pta*	MG1655, *mgsA*::M1-93-*fadZ*, Δ*fadE*, Δ*fumAC*, Δ*pta*	This study
Plasmids		
pJMYEEI82564 (TE10)	pTrc-EEI82564 thioesterase from *Anaerococcus tetradius*, Amp^r^	[49]

### Using OptForce for octanoic acid overproduction

OptForce optimization algorithm was used for identifying a prioritized intervention strategy for octanoate overproduction. The *i*AF1260 genome-scale metabolic (GSM) model in *E. coli* [63] was used for all the simulations under aerobic minimal conditions with glucose as the sole carbon substrate. A couple of modifications were made to the original GSM model (for example, activation of the beta-oxidation pathway reactions regulated off in the original model) based on prior experimental observations [13, 31]. Details of the modification, along with the flux bounds for exchange reactions for media metabolites, have been tabulated in Additional file 6: Text S1. Description of the sequential steps for implementation of the entire OptForce algorithm, along with details of the optimization formulations in each step, has been described elsewhere [31, 64]. In short, ^13^C-MFA for a wild-type *E. coli* strain [31] is imposed on the GSM model to characterize its native phenotype. This phenotype is contrasted to an overproduction phenotype producing octanoate at least 90% of its theoretical maximum capability (as well as a minimum growth-rate of 10% of its theoretical maximum). The contrast reveals a subset of reactions that “MUST” be altered (directly or indirectly) from their native activity to ensure octanoate overproduction. Note that the MUST set of reactions can be identified for increasing levels of complexity. At the “singles” level, individual reactions whose overproduction flux range has no overlap with the corresponding flux range in the native phenotype (either as an up-regulation or a down-regulation) are identified (i.e., MUST singles). The same analysis can be extended to identify pairs, triplets, or higher combination of reactions whose sum (or difference) of fluxes in the overproduction phenotype completely departs from the corresponding flux combination in the wild-type phenotype, even though their individual flux ranges have an overlap. This leads towards identification of MUST pairs, triples, and higher order sets. For the current simulation, the analysis as limited to doubles due to relative abundance of target reactions identified in the MUST sets. Finally, a bilevel optimization algorithm identifies a prioritized list of interventions from the MUST set of reactions that must be directly engineered to improve octanoate yield. See Additional file 6: Text S1 for expanded execution guidelines. The SBML file for iAF1260 model was obtained from the BIGG models repository (http://bigg.ucsd.edu/models/iAF1260). In addition, GAMS compatible files for the iAF1260 model and the OptForce code in GAMS for current simulation have been included in Additional file 7. Further description of the protocol can be found from http://maranasgroup.com/software.htm.

### In vivo production of C8 in shake flask

Individual colonies were selected from Luria Broth (LB) plates with ampicillin, inoculated into 3 mL of LB liquid medium with ampicillin for 4 h. Then, 0.5 mL of culture was added to 10 mL M9 (0.8 g/L NH_4_Cl, 0.5 g/L NaCl, 7.52 g/L Na_2_HPO_4_, 3.0 g/L KH_2_PO_4_, 0.24 g/L MgSO_4_, 11.1 mg/L CaCl_2_, 1 mg/L thiamine HCl, and trace elements containing 166.7 μg/L FeCl_3_·6H_2_O, 1.8 μg/L ZnSO_4_·7H_2_O, 1.2 μg/L CuCl_2_·2H_2_O, 1.2 μg/L MnSO_4_·2H_2_O, 1.8 μg/L CoCl_2_·6H_2_O, and 0.223 mg/L Na_2_EDTA·2H_2_O) with 1.5% (wt/v) dextrose at 30 °C, 220 rpm overnight for seed-culture preparation. Mid-log phase seed-culture was transferred into 40 mL M9 with 1.5% (wt/v) dextrose medium at the final concentration of OD_550_ ~ 0.1. For inducing *TE10* gene expression, isopropyl-β-d-thiogalactopyranoside (IPTG) was typically added at a final concentration of 1 mM when the OD_550_ reached 0.4–0.5. Cultures were grown in 250-mL baffled flasks with initial pH 7.0 at 30 °C, 220 rpm for 72 h. In M9 minimal medium, 0.8 g/L of ammonium chloride (NH_4_Cl) was used as the only nitrogen source, resulting in a standard C/N ratio of 18.8. For changing the C/N ratio, the glucose (carbon source) concentration was fixed at 15 g/L and the NH_4_Cl (carbon source) was added at final concentrations of 0.2, 0.4, 1.6, and 2.4 g/L, corresponding to C/N ratios of 75, 37.5, 9.4, and 6.25, respectively.

### In vivo production of C8 in bioreactor

Batch cultures were performed in 300 mL M9+ 1.5% (wt/v) dextrose in 500-mL bioreactor (INFORS HT). Cultures were grown at 30 °C, and the pH was maintained at 7.0 by adding 2.0 M potassium hydroxide (KOH). Air flow rate was initially maintained at 0.3 L/min. The dissolved oxygen (DO) level was set over 40% and controlled by changing the stirring speed, with a maximum value of 600 rpm. Similar operations were performed for fed-batch culture, the only difference being that a 9 mL of 50% (wt/v) glucose was fed to the cultures after 48-h cultivation.

### Determination of carboxylic acid titers

Carboxylic acid production was quantified by an Agilent 7890 gas chromatograph equipped with an Agilent 5975 mass spectroscope using mass spectrometer (GC–MS) after carboxylic acid extraction [17]. Briefly, 1 mL of culture was mixed with 125 μL of 10% (wt/v) sodium chloride and 125 μL of acetic acid. Then, 20 μL of 1 mg/mL internal standards (C9:0/C13:0/C15:0/C17:0) was added into the mixture followed by 500 μL of ethyl acetate. The mixture was vortexed for 30 s and then centrifuged at 16,000×*g* for 10 min. After that, 250 μL of top organic phase was transferred to a new glass tube followed by addition of 2.25 mL of ethanol:hydrochloric acid (30:1 v/v). After incubation at 55 °C for 1 h, 1.25 mL of double-distilled water (ddH_2_O) and 1.25 mL of hexane were added, vortexed, and centrifuged at 2000×*g* for 2 min. The top hexane layer was then transferred and analyzed by GC–MS. The temperature for GC–MS analysis was initially held at 50 °C for 2 min, ramped to 200 °C at 25 °C/min, held for 1 min, and then raised to 315 °C at 25 °C/min, held for 2 min. Helium was used as a carrier gas and the flow rate was set as 1 mL/min through a DB-5MS separation column (30 m, 0.25 mm ID, 0.25 μm, Agilent).

### Statistical analysis

The two-tailed *t* test method (two-sample equal variance, homoscedastic) was employed to analyze the statistical significance of all the data in this study and *P* value < 0.05 is deemed statistically significant.

## Additional files

**Additional file 1: Table S1.** The simulations predicted by the iAF1260 genome-scale metabolic model of *E. coli.*

**Additional file 2: Figure S1.** Schematic of overexpression of *fabZ, fabG, fabB/F* together for C8 production.

**Additional file 3: Figure S2.** Comparison of Δ*fumAC* alone and +*fabZ* Δ*fadE* Δ*fumAC* combination in cellular growth during C8 production.

**Additional file 4: Figure S3.** Effects of overexpression of *accABCD* on C8 production.

**Additional file 5: Table S2.** Primers and sequence used in this study.

**Additional file 6: Text S1.** Details of OptForce simulation for overproduction of octanoate in *E. coli*.

**Additional file 7.** GAMS compatible files for the iAF1260 model and the OptForce code.

## Abbreviations

SCFA: short-chain fatty acid
MCFA: medium-chain fatty acid
C8: octanoic acid
IPTG: isopropyl β-d-1-thiogalactopyranoside
ZEFA: +*fadZ* Δ*fadE* Δ*fumAC* Δ*ackA*
TE10: thioesterase from *Anaerococcus tetradius*

## References

[1] Larson ED (2006). A review of life-cycle analysis studies on liquid biofuel systems for the transport sector. Energy Sustain Dev.

[2] Gallezot P (2007). Process options for converting renewable feedstocks to bioproducts. Green Chem.

[3] Wymann MP, Schneiter R (2008). Lipid signalling in disease. Nat Rev Mol Cell Biol.

[4] Desbois AP, Smith VJ (2010). Antibacterial free fatty acids: activities, mechanisms of action and biotechnological potential. Appl Microbiol Biotechnol.

[5] Black BA, Zannini E, Curtis JM, Ganzle MG (2013). Antifungal hydroxy fatty acids produced during sourdough fermentation: microbial and enzymatic pathways, and antifungal activity in bread. Appl Environ Microbiol.

[6] Sokolsky-Papkov M, Shikanov A, Ezra A, Vaisman B, Domb AJ (2009). Fatty acid-based biodegradable polymers: synthesis and applications. Polymer Degrad Perform.

[7] Lopez-Ruiz JA, Davis RJ (2014). Decarbonylation of heptanoic acid over carbon-supported platinum nanoparticles. Green Chem.

[8] Lennen RM, Braden DJ, West RM, Dumesic JA, Pfleger BF (2010). A process for microbial hydrocarbon synthesis: overproduction of fatty acids in *Escherichia coli* and catalytic conversion to alkanes. Biotechnol Bioeng.

[9] Korstanje TJ, van der Vlugt JI, Elsevier CJ, de Bruin B (2015). Hydrogenation of carboxylic acids with a homogeneous cobalt catalyst. Science.

[10] Maki-Arvela P, Kubickova I, Snare M, Eranen K, Murzin DY (2007). Catalytic deoxygenation of fatty acids and their derivatives. Energy Fuels.

[11] Chatterjee A, Jensen VR (2017). A heterogeneous catalyst for the transformation of fatty acids to α-olefins. ACS Catal.

[12] Tee TW, Chowdhury A, Maranas CD, Shanks JV (2014). Systems metabolic engineering design: fatty acid production as an emerging case study. Biotechnol Bioeng.

[13] Steen EJ, Kang YS, Bokinsky G, Hu ZH, Schirmer A, McClure A, del Cardayre SB, Keasling JD (2010). Microbial production of fatty-acid-derived fuels and chemicals from plant biomass. Nature.

[14] Clomburg JM, Vick JE, Blankschien MD, Rodriguez-Moya M, Gonzalez R (2012). A synthetic biology approach to engineer a functional reversal of the beta-oxidation cycle. ACS Synth Biol.

[15] Liu H, Yu C, Feng DX, Cheng T, Meng X, Liu W, Zou HB, Xian M (2012). Production of extracellular fatty acid using engineered *Escherichia coli*. Microb Cell Fact.

[16] Lu XF, Vora H, Khosla C (2008). Overproduction of free fatty acids in *E. coli*: implications for biodiesel production. Metab Eng.

[17] Torella JP, Ford TJ, Kim SN, Chen AM, Way JC, Silver PA (2013). Tailored fatty acid synthesis via dynamic control of fatty acid elongation. Proc Natl Acad Sci USA.

[18] Xu P, Gu Q, Wang W, Wong L, Bower AG, Collins CH, Koffas MA (2013). Modular optimization of multi-gene pathways for fatty acids production in *E*. *coli*. Nat Commun.

[19] Zheng Y, Li L, Liu Q, Qin W, Yang J, Cao Y, Jiang X, Zhao G, Xian M (2012). Boosting the free fatty acid synthesis of *Escherichia coli* by expression of a cytosolic *Acinetobacter baylyi* thioesterase. Biotechnol Biofuels.

[20] Knothe G (2009). Improving biodiesel fuel properties by modifying fatty ester composition. Energy Environ Sci.

[21] Choi JW, Da Silva NA (2014). Improving polyketide and fatty acid synthesis by engineering of the yeast acetyl-CoA carboxylase. J Biotechnol.

[22] Park J, Rodriguez-Moya M, Li M, Pichersky E, San KY, Gonzalez R (2012). Synthesis of methyl ketones by metabolically engineered *Escherichia coli*. J Ind Microbiol Biotechnol.

[23] Wu H, Karanjikar M, San KY (2014). Metabolic engineering of *Escherichia coli* for efficient free fatty acid production from glycerol. Metab Eng.

[24] Zhu Z, Zhou YJ, Krivoruchko A, Grininger M, Zhao ZK, Nielsen J (2017). Expanding the product portfolio of fungal type I fatty acid synthases. Nat Chem Biol.

[25] Gajewski J, Pavlovic R, Fischer M, Boles E, Grininger M (2017). Engineering fungal de novo fatty acid synthesis for short chain fatty acid production. Nat Commun.

[26] Van Eerten-Jansen MCAA, Ter Heijne A, Grootscholten TIM, Steinbusch KJJ, Sleutels THJA, Hamelers HVM, Buisman CJN (2013). Bioelectrochemical production of caproate and caprylate from acetate by mixed cultures. ACS Sustain Chem Eng.

[27] Steinbusch KJJ, Hamelers HVM, Plugge CM, Buisman CJN (2011). Biological formation of caproate and caprylate from acetate: fuel and chemical production from low grade biomass. Energy Environ Sci.

[28] Spirito CM, Richter H, Rabaey K, Stams AJ, Angenent LT (2014). Chain elongation in anaerobic reactor microbiomes to recover resources from waste. Curr Opin Biotechnol.

[29] Jing FY, Cantu DC, Tvaruzkova J, Chipman JP, Nikolau BJ, Yandeau-Nelson MD, Reilly PJ (2011). Phylogenetic and experimental characterization of an acyl-ACP thioesterase family reveals significant diversity in enzymatic specificity and activity. BMC Biochem.

[30] Tan Z, Yoon JM, Nielsen DR, Shanks JV, Jarboe LR (2016). Membrane engineering via trans unsaturated fatty acids production improves *Escherichia coli* robustness and production of biorenewables. Metab Eng.

[31] Ranganathan S, Tee TW, Chowdhury A, Zomorrodi AR, Yoon JM, Fu Y, Shanks JV, Maranas CD (2012). An integrated computational and experimental study for overproducing fatty acids in *Escherichia coli*. Metab Eng.

[32] Xu P, Ranganathan S, Fowler ZL, Maranas CD, Koffas MAG (2011). Genome-scale metabolic network modeling results in minimal interventions that cooperatively force carbon flux towards malonyl-CoA. Metab Eng.

[33] Ranganathan S, Suthers PF, Maranas CD (2010). OptForce: an optimization procedure for identifying all genetic manipulations leading to targeted overproductions. PLoS Comput Biol.

[34] Jiang Y, Chen B, Duan CL, Sun BB, Yang JJ, Yang S (2015). Multigene editing in the *Escherichia coli* genome via the CRISPR–Cas9 system. Appl Environ Microbiol.

[35] Janssen HJ (2014). Fatty acid synthesis in *Escherichia coli* and its applications towards the production of fatty acid based biofuels. Biotechnol Biofuels.

[36] Wu G, Yan Q, Jones JA, Tang YJ, Fong SS, Koffas MAG (2016). Metabolic burden: cornerstones in synthetic biology and metabolic engineering applications. Trends Biotechnol.

[37] Li Q, Fan F, Gao X, Yang C, Bi C, Tang J, Liu T, Zhang X (2017). Balanced activation of IspG and IspH to eliminate MEP intermediate accumulation and improve isoprenoids production in *Escherichia coli*. Metab Eng.

[38] Yadav VG, De Mey M, Lim CG, Ajikumar PK, Stephanopoulos G (2012). The future of metabolic engineering and synthetic biology: towards a systematic practice. Metab Eng.

[39] Pitera DJ, Paddon CJ, Newman JD, Keasling JD (2007). Balancing a heterologous mevalonate pathway for improved isoprenoid production in *Escherichia coli*. Metab Eng.

[40] Tan Z, Chen J, Zhang X (2016). Systematic engineering of pentose phosphate pathway improves *Escherichia coli* succinate production. Biotechnol Biofuels.

[41] Ajikumar PK, Xiao WH, Tyo KE, Wang Y, Simeon F, Leonard E, Mucha O, Phon TH, Pfeifer B, Stephanopoulos G (2010). Isoprenoid pathway optimization for Taxol precursor overproduction in *Escherichia coli*. Science.

[42] Tseng CP, Yu CC, Lin HH, Chang CY, Kuo JT (2001). Oxygen- and growth rate-dependent regulation of *Escherichia coli* fumarase (FumA, FumB, and FumC) activity. J Bacteriol.

[43] Tseng CP (1997). Regulation of fumarase (*fumB*) gene expression in *Escherichia coli* in response to oxygen, iron and heme availability: role of the *arcA*, *fur*, and *hemA* gene products. FEMS Microbiol Lett.

[44] Li M, Zhang X, Agrawal A, San KY (2012). Effect of acetate formation pathway and long chain fatty acid CoA-ligase on the free fatty acid production in *E. coli* expressing acy-ACP thioesterase from *Ricinus communis*. Metab Eng.

[45] Baneyx F (1999). Recombinant protein expression in *Escherichia coli*. Curr Opin Biotechnol.

[46] Sriubolmas N, Panbangred W, Sriurairatana S, Meevootisom V (1997). Localization and characterization of inclusion bodies in recombinant *Escherichia coli* cells overproducing penicillin G acylase. Appl Microbiol Biotechnol.

[47] Zhang X, Li M, Agrawal A, San KY (2011). Efficient free fatty acid production in *Escherichia coli* using plant acyl-ACP thioesterases. Metab Eng.

[48] Singhasuwan S, Choorit W, Sirisansaneeyakul S, Kokkaew N, Chisti Y (2015). Carbon-to-nitrogen ratio affects the biomass composition and the fatty acid profile of heterotrophically grown *Chlorella* sp. TISTR 8990 for biodiesel production. J Biotechnol.

[49] Jarboe LR, Royce LA, Liu P (2013). Understanding biocatalyst inhibition by carboxylic acids. Front Microbiol.

[50] Yu X, Liu T, Zhu F, Khosla C (2011). In vitro reconstitution and steady-state analysis of the fatty acid synthase from *Escherichia coli*. Proc Natl Acad Sci USA.

[51] Lee S, Jung Y, Lee S, Lee J (2013). Correlations between FAS elongation cycle genes expression and fatty acid production for improvement of long-chain fatty acids in *Escherichia coli*. Appl Biochem Biotechnol.

[52] He L, Xiao Y, Gebreselassie N, Zhang F, Antoniewiez MR, Tang YJ, Peng L (2014). Central metabolic responses to the overproduction of fatty acids in *Escherichia coli* based on ^13^C-metabolic flux analysis. Biotechnol Bioeng.

[53] Liu D, Wan N, Zhang F, Tang YJ, Wu SG (2017). Enhancing fatty acid production in *Escherichia coli* by *Vitreoscilla* hemoglobin overexpression. Biotechnol Bioeng.

[54] Royce LA, Liu P, Stebbins MJ, Hanson BC, Jarboe LR (2013). The damaging effects of short chain fatty acids on *Escherichia coli* membranes. Appl Microbiol Biotechnol.

[55] Lennen RM, Pfleger BF (2013). Modulating membrane composition alters free fatty acid tolerance in *Escherichia coli*. PLoS ONE.

[56] San KY, Han S, Li W, Li M, Li Z. Fatty acids with mg transporter and mg. US Patent. 20150259712 A1.

[57] Sherkhanov S, Korman TP, Bowie JU (2014). Improving the tolerance of *Escherichia coli* to medium-chain fatty acid production. Metab Eng.

[58] Tan Z, Black W, Yoon JM, Shanks JV, Jarboe LR (2017). Improving *Escherichia coli* membrane integrity and fatty acid production by expression tuning of FadL and OmpF. Microb Cell Fact.

[59] Wu J, Zhang X, Xia X, Dong M (2017). A systematic optimization of medium chain fatty acid biosynthesis via the reverse beta-oxidation cycle in *Escherichia coli*. Metab Eng.

[60] Dellomonaco C, Clomburg JM, Miller EN, Gonzalez R (2011). Engineered reversal of the beta-oxidation cycle for the synthesis of fuels and chemicals. Nature.

[61] Kim S, Clomburg JM, Gonzalez R (2015). Synthesis of medium-chain length (C6–C10) fuels and chemicals via beta-oxidation reversal in *Escherichia coli*. J Ind Microbiol Biotechnol.

[62] Royce LA, Yoon JM, Chen Y, Rickenbach E, Shanks JV, Jarboe LR (2015). Evolution for exogenous octanoic acid tolerance improves carboxylic acid production and membrane integrity. Metab Eng.

[63] Feist AM, Henry CS, Reed JL, Krummenacker M, Joyce AR, Karp PD, Broadbelt LJ, Hatzimanikatis V, Palsson BO (2007). A genome-scale metabolic reconstruction for *Escherichia coli* K-12 MG1655 that accounts for 1260 ORFs and thermodynamic information. Mol Syst Biol.

[64] Chowdhury A, Zomorrodi AR, Maranas CD (2015). Bilevel optimization techniques in computational strain design. Comput Chem Eng.

