# Supplementary material for: Engineering of E. coli inherent fatty acid biosynthesis capacity to increase octanoic acid production
Source: Biotechnol Biofuels. 2018 Apr 2;11:87. doi: 10.1186/s13068-018-1078-z (PMC5879999; doi:10.1186/s13068-018-1078-z)
Supplement: Supplementary file 2 — Additional file 2: Figure S1. Schematic of overexpression of fabZ, fabG, fabB/F together for C8 production. [file 13068_2018_1078_MOESM2_ESM.docx]

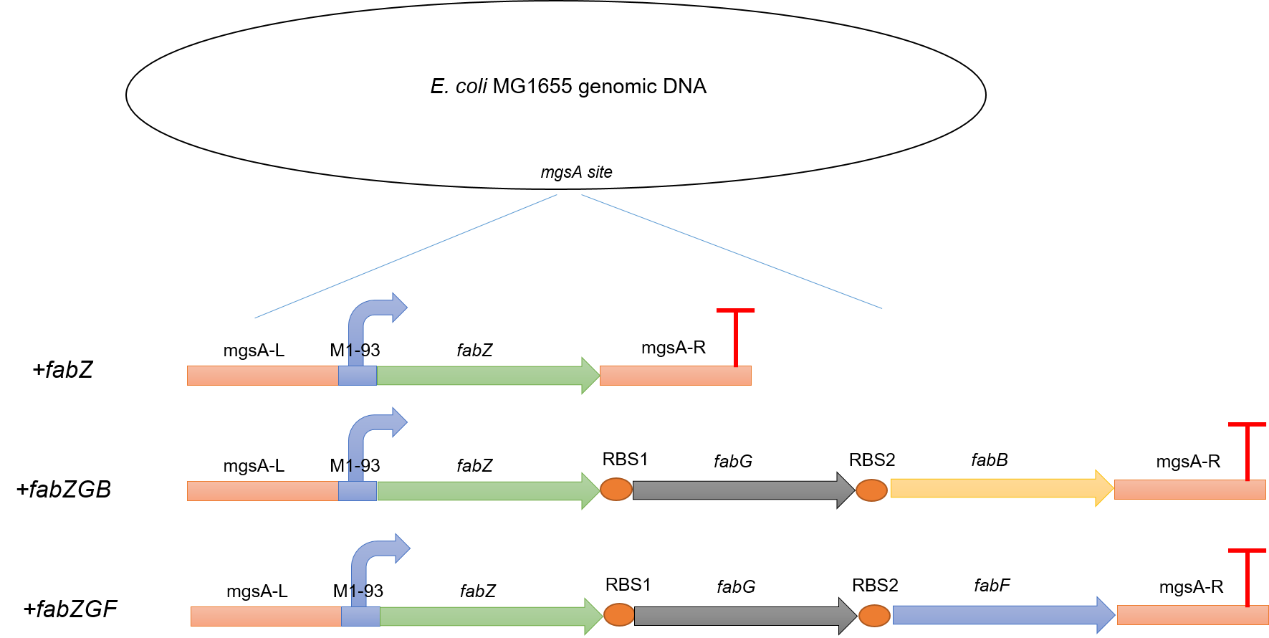


**Additional file 2: Figure S1. Schematic of overexpression of *fabZ*, *fabG*, *fabB/F* together for C8 production.**
