# Supplementary material for: Engineering of E. coli inherent fatty acid biosynthesis capacity to increase octanoic acid production
Source: Biotechnol Biofuels. 2018 Apr 2;11:87. doi: 10.1186/s13068-018-1078-z (PMC5879999; doi:10.1186/s13068-018-1078-z)
Supplement: Supplementary file 4 — Additional file 4: Figure S3. Effects of overexpression of accABCD on C8 production. [file 13068_2018_1078_MOESM4_ESM.docx]

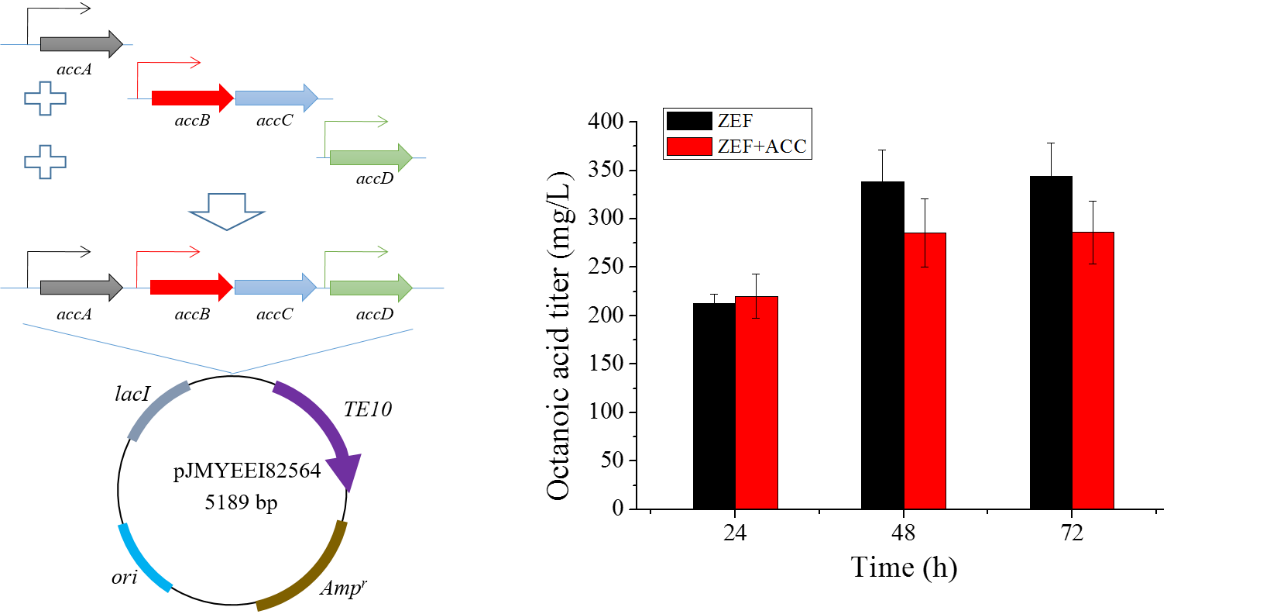


**Additional file 4: Figure S3. Effects of ACC overexpression on C8 production.** Left column shows the schematic of overexpression of acetyl-CoA carboxylase (AccABCD) from pJMYEEI82564 plasmid. Fermentations were performed in 40 mL M9+1.5% (wt/v) dextrose in 250 mL shake flasks at 250 rpm 30 °C with an initial pH of 7.0, IPTG of 1 mM. Titers are the average of at least three biological replicates at 72 h with error bars indicating one standard deviation. ZEF, *+fadZ* Δ*fadE* Δ*fum.*
