# Supplementary material for: Engineering of E. coli inherent fatty acid biosynthesis capacity to increase octanoic acid production
Source: Biotechnol Biofuels. 2018 Apr 2;11:87. doi: 10.1186/s13068-018-1078-z (PMC5879999; doi:10.1186/s13068-018-1078-z)
Supplement: Supplementary file 6 — Additional file 6: Text S1. Details of OptForce simulation for overproduction of octanoate in E. coli. [file 13068_2018_1078_MOESM6_ESM.docx]

Additional file 6: Text S1. Details of OptForce simulation for overproduction of octanoate in *E. coli*

1. Details of the optimization simulation
2. GSM model: *i*AF1260 model of *E. coli* MG1655. The SBML file for the model is available in BIGG models database (<http://bigg.ucsd.edu/models/iAF1260>). See Supplementary zipped attachment F1 for GAMS compatible files for the model.
3. Growth media and fermentation: Minimal M9 media with glucose as the sole limiting carbon substrate. Fermentation under aerobic conditions
4. Optimization algorithm: The OptForce optimization algorithm (cite <http://www.sciencedirect.com/science/article/pii/S0098135414001860>) was used for integrating 13C-MFA information for the wild-type *E. coli* phenotype, and identifying a prioritized genetic intervention strategy for overproduction of octanoate. See Supplementary zipped attachment F1 for for full implementation of the algorithm in GAMS). Further details of using the OptForce algorithm are available in the Maranas group webpage (<http://maranasgroup.com/software.htm>).
5. Summary of the OptForce procedural steps:
6. Characterize the wild-type and overproduction phenotypes:
7. Run MaxBiomass.gms: Using the flux bounds on central carbon reactions obtained for 13C-MFA (included in file iAF1260_MFA_bounds.txt), maximize the growth rate of representative biomass reaction (Ec_biomass_iAF1260_WT_59p81M) in the model
8. Run BoundsWT.gms: Add the constraint on biomass obtained in (a) to the 13C-MFA bounds, and sequentially maximize and minimize the flux of each reaction in the model.
9. Run MaxTarget.gms: Maximize the exchange flux for octanoate (EX_octa(e)) with no constraint on biomass
10. Run BoundsON.gms: Fix octanoate exchange flux to 90% of that obtained from ( c), and biomass to 10% for its theoretical maximum, and sequentially maximize and minimize the flux of each reaction
11. Find single-level MUST sets:
12. Run MustU.gms: Find all reactions whose overproduction flux bound is higher than the corresponding flux bound in wild-type phenotype
13. Run MustL.gms: Find all reactions whose overproduction flux bound is higher than the corresponding flux bound in wild-type phenotype
14. Run MustOnes.py: Collect together all the single-level Must set reactions
15. Find second-level MUST sets:
16. Run MustUU.gms: Find all pairs of reactions whose overproduction flux bound sum is higher than the corresponding sum in wild-type phenotype
17. Run MustLL.gms: Find all pairs of reactions whose overproduction flux bound sum is higher than the corresponding sum in wild-type phenotype
18. Run MustUL.gms: Find all pairs of reactions whose overproduction flux bound difference is higher than the corresponding difference in wild-type phenotype
19. Run MustForce.py: Collect together first and second-level Must sets, and separate them into buckets of up-regulation, down-regulation, or removal.
20. Find the FORCE sets:

a. Run OptForce.gms: Find the prioritized list of k interventions that would overproduce octanoate flux for a biomass flux at-least 10% of its theoretical maximum.

1. Modifications to *i*AF1260 GSM model to perform OptForce simulations for octanoate overproduction
2. Table of additional reactions included in the model

| id | name | equation | Lower bound (mmol gDW-1 h-1 | Upper bound (mmol gDW-1 h-1) | rxntype |
| --- | --- | --- | --- | --- | --- |
| OCTAtpp | octanoate periplasmic transport | octa[c] + h[c] -> octa[p] + h[p] | 0 | 1000 | 0 |

1. Table of regulated reactions (under glucose aerobic conditions) in the base model re-activated for current simulation

| id | name | equation | gpr | ec | lower bound (mmol gDW-1 h-1) | upper bound (mmol gDW-1 h-1) | rxntype |
| --- | --- | --- | --- | --- | --- | --- | --- |
| ACOAD1f_f | Acyl CoA dehydrogenase (butanoylCoA) | btcoa[c] + fad[c] -> b2coa[c] + fadh2[c] | FadE | 1.3.99.2 | -1000 | 1000 | 1 |
| ACOAD2f_f | Acyl CoA dehydrogenase (hexanoylCoA) | hxcoa[c] + fad[c] -> hx2coa[c] + fadh2[c] | FadE | 1.3.99.3 | -1000 | 1000 | 1 |
| ACOAD3f_f | Acyl CoA dehydrogenase (octanoylCoA) | occoa[c] + fad[c] -> oc2coa[c] + fadh2[c] | FadE | 1.3.99.3 | -1000 | 1000 | 1 |
| ECOAH1_f | 3-hydroxyacyl CoA dehydratase (3hydroxybutanoylCoA) | 3hbcoa[c] <=> b2coa[c] + h2o[c] | (FadB) or (FadJ) | 4.2.1.17 | -1000 | 1000 | 1 |
| ECOAH2_f | 3-hydroxyacyl CoA dehydratase (3hydroxyhexanoylCoA) | 3hhcoa[c] <=> hx2coa[c] + h2o[c] | (FadB) or (FadJ) | 4.2.1.17 | -1000 | 1000 | 1 |
| ECOAH3_f | 3-hydroxyacyl CoA dehydratase (3hydroxyoctanoylCoA) | 3hocoa[c] <=> oc2coa[c] + h2o[c] | (FadB) or (FadJ) | 4.2.1.17 | -1000 | 1000 | 1 |
| HACD1i | 3-hydroxyacyl CoA dehydrogenase (acetoacetylCoA) | 3hbcoa_c + nad[c] -> aacoa[c] + h[c] + nadh[c] | (FadB) or (FadJ) | 1.1.1.35 | 0 | 1000 | 0 |
| HACD2i | 3-hydroxyacyl CoA dehydrogenase (3oxohexanoylCoA) | 3hhcoa_c + nad[c] -> 3ohcoa[c] + h[c] + nadh[c] | (FadB) or (FadJ) | 1.1.1.35 | 0 | 1000 | 0 |
| HACD3i | 3-hydroxyacyl CoA dehydrogenase (3oxooctanoylCoA) | 3hocoa_c + nad[c] -> 3oocoa[c] + h[c] + nadh[c] | (FadB) or (FadJ) | 1.1.1.35 | 0 | 1000 | 0 |
| KAT1 | 3-ketoacyl CoA thiolase | aacoa[c] + coa[c] -> 2 accoa[c] | (FadI) or (FadA) | 2.3.1.16 | 0 | 1000 | 0 |
| KAT2 | 3-ketoacyl CoA thiolase | 3ohcoa[c] + coa[c] -> accoa[c] + btcoa[c] | (FadI) or (FadA) | 2.3.1.16 | 0 | 1000 | 0 |
| KAT3 | 3-ketoacyl CoA thiolase | 3oocoa[c] + coa[c] -> accoa[c] + hxcoa[c] | (FadI) or (FadA) | 2.3.1.16 | 0 | 1000 | 0 |
| ICL | Isocitrate lyase | icit[c] -> glx[c] + succ[c] | AceA | 4.1.3.1 | 0 | 1000 | 0 |
| MALS | Malate synthase | accoa[c] + glx[c] + h2o[c] -> coa[c] + h[c] + mal_L[c] | (AceB) or (GlcB) | 4.1.3.2 | 0 | 1000 | 0 |

1. Table of flux bounds on exchange reactions for media metabolites

| id | name | equation | lower bound (mmol gDW-1 h-1) | upper bound (mmol gDW-1 h-1) | rxntype |
| --- | --- | --- | --- | --- | --- |
| EX_glc(e) | Glucose exchange | glc_D[e] <=> | -100 | 1000 | 4 |
| EX_o2(e) | Oxygen exchange | o2[e] <=> | -200 | 1000 | 4 |
| EX_ca2(e) | Calcium exchange | ca2[e] <=> | -1000 | 1000 | 4 |
| EX_cl(e) | Chloride exchange | cl[e] <=> | -1000 | 1000 | 4 |
| EX_co2(e) | CO2 exchange | co2[e] <=> | -1000 | 1000 | 4 |
| EX_cobalt2(e) | Cobalt exchange | cobalt2[e] <=> | -1000 | 1000 | 4 |
| EX_cu2(e) | Copper exchange | cu2[e] <=> | -1000 | 1000 | 4 |
| EX_fe2(e) | Fe2 exchange | fe2[e] <=> | -1000 | 1000 | 4 |
| EX_fe3(e) | Fe3 exchange | fe3[e] <=> | -1000 | 1000 | 4 |
| EX_h(e) | proton exchange | h[e] <=> | -1000 | 1000 | 4 |
| EX_h2o(e) | H2O exchange | h2o[e] <=> | -1000 | 1000 | 4 |
| EX_k(e) | K exchange | k[e] <=> | -1000 | 1000 | 4 |
| EX_mg2(e) | Mg exchange | mg2[e] <=> | -1000 | 1000 | 4 |
| EX_mn2(e) | Mn2 exchange | mn2[e] <=> | -1000 | 1000 | 4 |
| EX_mobd(e) | Molybdate exchange | mobd[e] <=> | -1000 | 1000 | 4 |
| EX_na1(e) | Sodium exchange | na1[e] <=> | -1000 | 1000 | 4 |
| EX_nh4(e) | Ammonia exchange | nh4[e] <=> | -1000 | 1000 | 4 |
| EX_pi(e) | Phosphate exchange | pi[e] <=> | -1000 | 1000 | 4 |
| EX_so4(e) | Sulfate exchange | so4[e] <=> | -1000 | 1000 | 4 |
| EX_tungs(e) | tungstate exchange | tungs[e] <=> | -1000 | 1000 | 4 |
| EX_zn2(e) | Zinc exchange | zn2[e] <=> | -1000 | 1000 | 4 |
| EX_cbl1(e) | Cobalamine exchange | cbl1[e] <=> | -1000 | 1000 | 4 |

1. Table of flux bounds on central carbon metabolism reactions estimated from ^13^C MFA experimental data (cite PMID: 23036703) for wild-type phenotype simulation

| id | name | equation | gpr | ec | lower bound (mmol gDW-1 h-1) | upper bound (mmol gDW-1 h-1) | rxntype |
| --- | --- | --- | --- | --- | --- | --- | --- |
| PGI_f | glucose-6-phosphate isomerase | g6p[c] <=> f6p[c] | Pgi | 5.3.1.9 | 87.6629 | 88.7348 | 1 |
| FBA_f | fructose bisphosphate aldolase | fdp[c] <=> dhap[c] + g3p[c] | (FbaB) or (B1773) or (FbaA) | 4.1.2.13 | 88.4965 | 89.9803 | 1 |
| TPI_f | triose phosphate isomerase | dhap[c] <=> g3p[c] | Tpi | 5.3.1.1 | 72.2546 | 96.2449 | 1 |
| PGK_f | phosphoglycerate kinase | 3pg[c] + atp[c] <=> 13dpg[c] + adp[c] | Pgk | 2.7.2.3 | -180.6662 | -157.229 | 1 |
| ENO | enolase | 2pg[c] <=> h2o[c] + pep[c] | Eno | 4.2.1.11 | 141.0537 | 174.0194 | 1 |
| PYK | pyruvate kinase | adp[c] + h[c] + pep[c] -> atp[c] + pyr[c] | (Pyka) or (Pykf) | 2.7.1.40 | 49.0167 | 51.6513 | 0 |
| EDA | 2-dehydro-3-deoxy phosphogluconate aldolase | 2ddg6p[c] -> g3p[c] + pyr[c] | Eda | 4.1.2.14 | 1.4055 | 3.5058 | 0 |
| G6PDH2r_f | glucose-6-phosphate dehydrogenase | g6p[c] + nadp[c] <=> 6pgl[c] + h[c] + nadph[c] | Zwf | 1.1.1.49 | 10.1408 | 11.144 | 1 |
| GND | phosphogluconate dehydrogenase | 6pgc[c] + nadp[c] -> co2[c] + nadph[c] + ru5p_D[c] | Gnd | 1.1.1.44 | 7.4764 | 8.8971 | 0 |
| TALA_f | transaldolase | g3p[c] + s7p[c] <=> e4p[c] + f6p[c] | ( TalA ) or ( TalB ) | 2.2.1.2 | 1.6489 | 1.9671 | 1 |
| TKT1_f | transketolase | r5p[c] + xu5p_D[c] <=> g3p[c] + s7p[c] | (TktA) or (TktB) | 2.2.1.1 | 1.6489 | 1.9671 | 1 |
| TKT2_f | transketolase | e4p[c] + xu5p_D[c] <=> f6p[c] + g3p[c] | (TktA) or (TktB) | 2.2.1.1 | -15.8512 | 1.9132 | 1 |
| MDH_f | malate dehydrogenase | mal_L[c] + nad[c] <=> h[c] + nadh[c] + oaa[c] | Mdh | 1.1.1.37 | 45.1001 | 49.1938 | 1 |
| FUM_f | fumarase | fum[c] + h2o[c] <=> mal_L[c] | (FumA) or (FumB) or (FumCec) | 4.2.1.2 | 43.698 | 47.7271 | 1 |
| ICDHyr_f | isocitrate dehydrogenase (NADP) | icit[c] + nadp[c] <=> akg[c] + co2[c] + nadph[c] | Icd | 1.1.1.42 | 47.987 | 53.0088 | 1 |
| AKGDH | 2-Oxogluterate dehydrogenase | akg[c] + coa[c] + nad[c] -> co2[c] + nadh[c] + succoa[c] | (LpdA and SucAec and SucBec) | | 41.6785 | 46.5884 | 0 |
| ICL | Isocitrate lyase | icit[c] -> glx[c] + succ[c] | AceA | 4.1.3.1 | 0.6581 | 2.5001 | 0 |
| MALS | malate synthase | accoa[c] + glx[c] + h2o[c] -> coa[c] + h[c] + mal_L[c] | (AceB) or (GlcB) | 4.1.3.2 | 0.6581 | 2.5001 | 0 |
| MALDDH | malate decarboxylating oxidoreductase | mal_D[c] + nad[c] -> co2[c] + nadh[c] + pyr[c] | YeaU | 1.1.1.83 | 0 | 0.4776 | 0 |
| PGCD | phosphoglycerate dehydrogenase | 3pg[c] + nad[c] -> 3php[c] + h[c] + nadh[c] | SerA | 1.1.1.95 | 4.9571 | 14.3007 | 0 |
| GHMT2r_f | glycine hydroxymethyltransferase | ser_L[c] + thf[c] <=> gly[c] + h2o[c] + mlthf[c] | GlyA | 2.1.2.1 | 0.2895 | 10.4352 | 1 |
| SERD | L-serine deaminase | ser_L[c] -> nh4[c] + pyr[c] | (TdcG) or (SdaAec) or (SdaB) or (TnaA) | 4.3.1.17 | 0.8836 | 1.9676 | 0 |
| THRS | threonine synthase | h2o[c] + phom[c] -> pi[c] + thr_L[c] | ThrC | 4.2.3.1 | 3.8832 | 5.0902 | 0 |
| THRAi | Threonine aldolase | thr_L[c] -> acald[c] + gly[c] | (GlyA) or (LtaE) | 4.1.2.5 | 0 | 5.0902 | 0 |
| GLUDy_f | glutamate synthase (NADP) | akg[c] + gln_L[c] + h[c] + nadph[c] -> 2 glu_L[c] + nadp[c] | GltB | 1.4.1.13 | -10.3322 | 5.2647 | 0 |
| GLUSy | glutamate dehydrogenase (NADP) | glu_L[c] + h2o[c] + nadp[c] <=> akg[c] + h[c] + nadph[c] + nh4[c] | GdhA | 1.4.1.4 | 11.03 | 62.0917 | 1 |
| ASPTA_f | aspartate transaminase | akg[c] + asp_L[c] <=> glu_L[c] + oaa[c] | AspC | 2.6.1.1 | -23.6803 | -0.3907 | 1 |
| ALATA_L2 | L-alanine transaminase | akg[c] + ala_L[c] <=> glu_L[c] + pyr[c] | | 2.6.1.2 | 0 | 0.5645 | 1 |
| EX_ac(e) | Acetate exchange | ac[e] <=> |  |  | 64.7121 | 65.4821 | 4 |
| EX_co2(e) | CO2 exchange | co2[e] <=> |  |  | 218.0465 | 228.4775 | 4 |
| PPC | phosphoenolpyruvate carboxylase | co2[c] + h2o[c] + pep[c] -> h[c] + oaa[c] + pi[c] | Ppc | 4.1.1.31 | 18.4966 | 28.0904 | 0 |
| ACKr_f | acetate kinase | ac[c] + atp[c] <=> actp[c] + adp[c] | (TdcD) or (AckA) or (PurT) | 2.7.2.1 | -65.4821 | -62.5454 | 1 |
| PTAr_f | phosphotransacetylase | accoa[c] + pi[c] <=> actp[c] + coa[c] | (Pta) or (EutD) | 2.3.1.8 | 62.5454 | 65.4821 | 1 |
| Ec_biomass_iAF1260_WT_59p81M | cellular biomass | |  |  | 5 | 1000 | 0 |
